# Heterogeneity of MGMT expression in corticotroph adenomas and mechanism of acquired temozolomide resistance

**DOI:** 10.1210/clinem/dgag170

**Published:** 2026-05-18

**Authors:** Ticiana Paes, Richard A Feelders, Fadime Dogan, Anand M Iyer, Johan M Kros, Yair Schwarz, Peter M van Koetsveld, Leo J Hofland

**Affiliations:** Department of Internal Medicine, Division of Endocrinology, Erasmus MC Cancer Institute, Erasmus Medical Center, Rotterdam 3015 GD, The Netherlands; Department of Internal Medicine, Roger Williams Medical Center, Providence, RI 02908, USA; Department of Internal Medicine, Division of Endocrinology, Erasmus MC Cancer Institute, Erasmus Medical Center, Rotterdam 3015 GD, The Netherlands; Holman Division of Endocrinology, Diabetes and Metabolism, Department of Medicine, NewYork University Langone Medical Center, New York, NY 10016, USA; Department of Internal Medicine, Division of Endocrinology, Erasmus MC Cancer Institute, Erasmus Medical Center, Rotterdam 3015 GD, The Netherlands; Department of Internal Medicine, Division of Endocrinology, Erasmus MC Cancer Institute, Erasmus Medical Center, Rotterdam 3015 GD, The Netherlands; Department of Pathology, Erasmus Medical Center, Rotterdam 3015 GD, The Netherlands; Dina Recanati School of Medicine, Reichman University, Herzliyya 4610101, Israel; Division of Endocrinology, Diabetes and Metabolism, Sheba Medical Center, Ramat Gan 52621, Israel; Department of Internal Medicine, Division of Endocrinology, Erasmus MC Cancer Institute, Erasmus Medical Center, Rotterdam 3015 GD, The Netherlands; Department of Internal Medicine, Division of Endocrinology, Erasmus MC Cancer Institute, Erasmus Medical Center, Rotterdam 3015 GD, The Netherlands

**Keywords:** temozolomide, resistance, pituitary tumors, aggressive tumors, Cushing disease

## Abstract

**Context:**

Temozolomide (TMZ) can be an effective medical treatment for aggressive pituitary tumors. In case of disease recurrence following TMZ treatment, however, treatment with the drug generally does not control tumor regrowth.

**Objective:**

This work aimed to better understand the mechanisms of resistance of corticotroph tumors to TMZ in the context of heterogeneity of methylguanine-DNA methyltransferase (MGMT) expression.

**Methods:**

We performed immunohistochemical analysis of the MGMT expression pattern in 25 corticotroph tumors to evaluate intratumoral heterogeneity. In addition, we created in vitro models of AtT20 corticotroph tumor cells with acquired TMZ-resistance after exposure to high- and low-dose TMZ, the latter representing a clinically achievable TMZ level.

**Results:**

MGMT immunostaining in corticotroph tumors showed a considerable heterogeneous intertumoral and intratumoral distribution pattern in 80% of tumors. In the in vitro model, high- and low-dose TMZ challenges induced a 6.3- and 3.4-fold decreased sensitivity to the growth inhibitory effect of TMZ. TMZ-induced changes in cell cycle phases were lower in TMZ-resistant cells than in vehicle-challenged cells. TMZ-resistant cells had higher *Mgmt* messenger RNA and protein expression and 1.8-fold higher number of Mgmt-positive cells. No difference was observed in the level of *Mgmt* promoter methylation.

**Conclusion:**

Corticotroph pituitary tumors demonstrate a high intertumoral and intratumoral heterogeneity in MGMT expression. In an acquired TMZ-resistant corticotroph pituitary tumor cell model, TMZ resistance was associated with strong increase in MGMT expression and percentage of MGMT-positive cells. We hypothesize that clonal selection of high MGMT-expressing cells is involved in this acquired TMZ resistance.

Temozolomide (TMZ) can be used in aggressive pituitary tumors, showing tumor regression or stabilization in approximately 70% of cases (1-3). In corticotroph tumors, tumor regression has been reported in 38%, while stabilization of tumor progression is observed in 27% of cases (4). After an initial positive response to TMZ, a substantial number of the cases recur over time. According to guidelines for the management of aggressive pituitary tumors, in case of tumor progression after an initial response to TMZ, rechallenge with TMZ is considered an option. This recommendation is based on a compilation of patients who underwent a second course of TMZ, of whom 58% showed a partial response/tumor stabilization (5, 6). However, after recurrence, the second course of TMZ is usually less effective compared to the first course (1, 3, 4, 7-9).

The antineoplastic effect of TMZ is primarily attributed to DNA alkylation/methylation of DNA guanine at the O6 position, generating O6 methyl guanine (*O6*-MeG) residues, which mispair with thymine DNA bases (instead of cytosine). Failure of the DNA mammalian mismatch repair (MMR) to repair this mismatch results in DNA single- and double-strand breaks and triggers apoptosis (2, 10). Thus, an intact MMR system is critical for the antineoplastic effect of TMZ. On the contrary, a defective or nonfunctional MMR system leads to accumulation of TMZ-induced DNA damage without triggering cell death pathways, resulting in resistance to TMZ (1, 10-15).

Another important mechanism of TMZ resistance involves methylguanine-DNA methyltransferase (MGMT) expression. The methyl group added by TMZ, forming *O6*-MeG residues, can be removed through MGMT activity, thus reversing the cytotoxic effect of TMZ (12, 16). Therefore, high MGMT expression in tumors may result in TMZ resistance (1, 5, 17, 18). Adding one additional layer to the already complex mechanism of resistance, a heterogeneous pattern with subclones of cells exhibiting different levels of MGMT expression has been reported in pituitary tumors (19-21). Thus, we hypothesize that TMZ treatment can induce selection pressure, resulting in the emergence of clones of cells with a high MGMT expression phenotype, conferring secondary TMZ resistance.

The present study aimed to better understand the mechanism of sensitivity or resistance of corticotroph tumors to TMZ. First, we aimed to establish the pattern of MGMT expression in corticotroph tumors, focusing on intertumoral and intratumoral heterogeneity. Secondly, we created an in vitro model of acquired resistance to TMZ in a corticotroph pituitary tumor cell line and evaluated the mechanisms involved.

## Materials and methods

### Study cohort

Twenty-five patients who underwent surgery for Cushing Disease (CD) at Erasmus Medical Center were included. A medical record review was performed to collect clinical data, including sex, age of diagnosis, tumor size, CD severity, and disease remission after surgery. CD severity was based on the upper limit of normal (ULN) of 24-hour urinary free cortisol (UFC) concentrations. Mild CD was defined as a UFC level less than 2 times the ULN; moderate to severe CD as a UFC level above 2 times the ULN. Tumor size was determined before surgery and categorized as microadenoma (<1 cm) or macroadenoma (≥1 cm). Aggressiveness was defined as significant tumor growth despite optimal medical therapy (surgery, radiotherapy [RT], medical treatment) (5). The persistence of CD after surgery was defined as failure to induce biochemical remission according to UFC levels. Tumor invasiveness was based on magnetic resonance imaging showing cavernous sinus invasion.

The diagnosis of CD was based first on first-line screening tests, which included a dexamethasone suppression test, UFC, and late-night saliva cortisol concentrations (22, 23). Subsequently, the pituitary source of hypercortisolism was investigated, including measuring plasma adrenocorticotropin (ACTH) levels, magnetic resonance imaging, and, if needed, bilateral petrosal sinus sampling to detect pituitary tumors (22).

Permission from the institutional review board of the Erasmus MC was obtained. Informed consent was obtained to use the tumor tissues for research purposes. The study was performed retrospectively and according to the guidelines of the Central Committee on Research Involving Human Subjects.

### Methylguanine-DNA methyltransferase immunohistochemistry on corticotroph adenomas

Immunohistochemistry (IHC) was performed on 25 formalin-fixed, paraffin-embedded corticotroph tumor tissues on an accredited automated slide stainer (Benchmark Ultra System, Ventana Medical Systems). Following deparaffinization and heat-induced antigen retrieval, the tissue samples were incubated with the primary MGMT antibody (RRID:AB_831546; Supplementary Table S3), followed by Optiview detection (No. 760-700, Ventana) (24).

Counterstain was performed using hematoxylin II and a blue coloring reagent. Each tissue slide contained a fragment of formalin-fixed, paraffin-embedded colon and tonsil as an on-slide positive control. In all cases analyzed, including the negative cases, the positive control was strongly positive. MGMT expression was considered positive only when nuclear immunostaining was observed. The level of MGMT protein expression was assessed independently by 2 investigators (L.J.H. and T.P.) and scored on a 4-tiered scale as follows: 0: negative, 1: 1% to 25%, 2: 26% to 50%, 3: 51% to 75%, and 4: more than 75% positive cells, according to the fraction of positive nuclear immunostaining. Discordant results between investigators were discussed to reach a final consensus. In 1 tumor, 2 completely distinct parts were observed; therefore, this specimen received 2 different classifications.

### Cell culture and compounds

The mouse corticotroph pituitary adenoma cell line (AtT20) was used (ATCC; CRL-1795). ATt20 cells were passaged and cultured in Dulbecco's Minimal Essential Medium supplemented with fetal calf serum 10%, penicillin, and glutamine and incubated in a 75-cm^2^ flask at 37 °C under standard conditions. The cells were routinely passaged by trypsinization with trypsin (0.05%)-EDTA (0.53 mM) every 7 days, as needed for the experiment and with a limited number of 18 passages in total, as described previously (25).

After trypsinization, the cells were plated in 24-well plates at adequate density to achieve 80% of growth confluence at the end of the experiment. After 1 day, a 7-day incubation with increasing concentrations of TMZ (range, 1-500 µM) or vehicle was started. Medium and drugs were refreshed at day 3. To evaluate the effect of TMZ on cell growth, we determined DNA concentration per well (measure of cell number), using the Hoechst 33258 method (26).

TMZ was purchased from Sigma-Aldrich, dissolved in 100% of dimethyl sulfoxide (DMSO) to prepare stock solution of 100 mM, and stored at −20 °C.

### Generation of temozolomide-resistant cells

The protocols consisted of challenging the AtT20 cells with TMZ at specified concentrations for 24 hours, after which medium was changed with medium without TMZ. After 6 days without drug exposure, the cells were trypsinized. Controls included treatment with challenges of the vehicle DMSO (1% final concentration).

Two protocols were used, one based on a high dose of TMZ and another using lower TMZ concentrations that are clinically achievable after a standard TMZ schedule (150-200 mg/day/m^2^ for 5 days; peak plasma concentration 50-72 µM) (5, 27, 28).

The high TMZ challenge (high TMZ_ch) protocol consisted of 3 pulse cycles of 24-hour exposure to increasing concentrations of TMZ. Initially, the cells were challenged with TMZ at a dose of 100 µM for 24 hours. Then, the cells were cultured in drug-free media for 4 weeks to achieve appropriate growth and proliferation. Subsequently, the cells were challenged with TMZ at a concentration of 200 µM for 24 hours then cultured for 3 weeks in drug-free media. The last challenge consisted of TMZ dose of 300 µM. After the third challenge, the resistant cells had grown for 9 passages with no drug treatment.

The low TMZ challenge (low TMZ_ch) protocol consisted of 4 pulse cycles of 24-hour exposure to increasing concentrations of TMZ with 2-week intervals between them. Initially, the cells were challenged with TMZ at a dose of 20 µM for 24 hours. The following reexposures to TMZ were performed in the same manner with TMZ concentrations of 40 µM (second challenge), 60 µM (third challenge), and 80 µM (fourth challenge). Subsequently, the resistant cells and their counterparts (vehicle-challenged cells) were cultured for an additional 9 weeks with no drug treatment. Treatment schedules are shown in Fig. 1.

**Figure 1:**
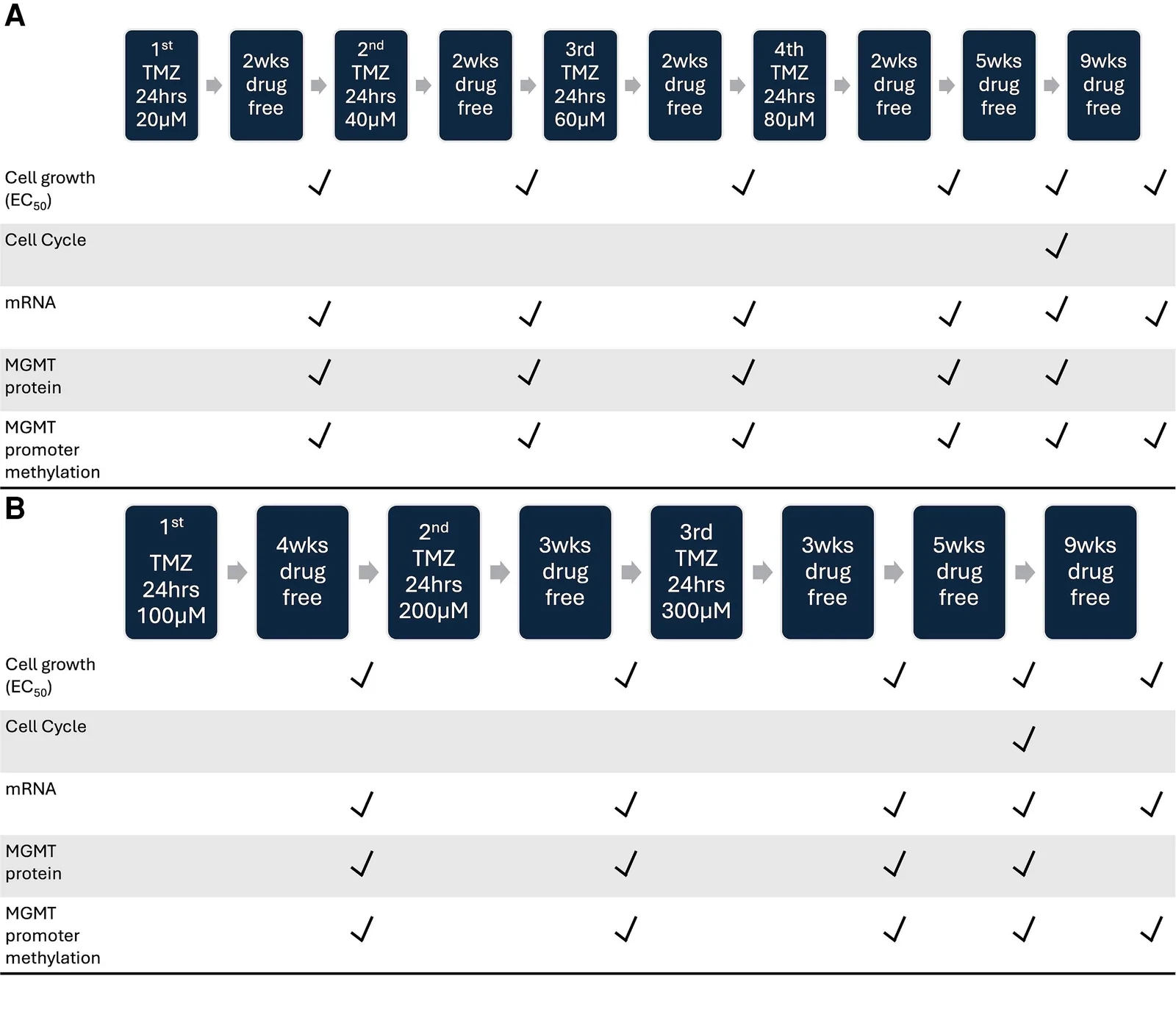
Treatment schedules. A, Treatment schedule for the high temozolomide (TMZ)-challenge protocol; B, treatment schedule for the low TMZ-challenge protocol.

For both high TMZ_ch and low TMZ_ch protocols, resistant cells and vehicle-challenged control cells were cultured for an additional 9 weeks with no drug treatment.

Following each TMZ/vehicle exposure, dose-response experiments were performed to determine cell sensitivity to TMZ, consisting of a 7-day incubation with increasing TMZ concentrations. The TMZ concentrations ranged from 1 to 500 µM: 500 µM, 100 µM, 75 µM, 50 µM, 37.5 µM, 25 µM, 10 µM, and 1 µM. After 5- and 9-week drug-free periods following the last TMZ challenge, dose-response experiments were performed as well. This was done to confirm that TMZ-resistant cells were stable.

### Cell cycle analysis

To characterize a resistant cell phenotype, cell cycle analysis was performed. Cells from the high TMZ_ch protocol, 5 weeks after the third TMZ or vehicle (DMSO) challenge, were treated with vehicle (DMSO) or TMZ at the dose of 100 µM under standard culture conditions. For the low TMZ_ch protocol, 5 weeks after the fourth TMZ or vehicle (DMSO) challenge, cells were treated with vehicle (DMSO) or TMZ at the dose of 80 µM. After 3 days, the cells were collected and washed twice with phosphate-buffered saline. Subsequently, the cells were fixed with ice-cold 70% ethanol and stored at −20 °C for less than 1 week. The cell cycle analysis was performed using Muse Cell Cycle reagent and Muse Cell Analyzer (Merck Millipore).

### Messenger RNA expression analysis

*Mgmt* and MMR components (*Msh2*, *Msh6*, *Mlh1*, and *Pms2*) messenger RNA (mRNA) expression was assessed in vehicle- and TMZ-challenged AtT20 cells at indicated time points.

Briefly, we isolated RNA with a High Pure RNA Isolation Kit (Roche Diagnostic GmbH), according to the manufacturer's protocol. Afterward, complementary DNA was synthesized using the commercial RevertAid First Strand cDNA synthesis kit (Thermo Scientific) and quantitative polymerase chain reaction (qPCR) was performed as previously described (29). Reference genes for relative quantification included *Hprt*, *Gusb*, and *Gapdh*. Primer-probe information can be found in Supplementary Table S2 (24). Data analysis was performed using the QuantStudio 7 Flex RT-qPCR system with QuantStudio Real-Time PCR software v1.5. The relative expression of genes was calculated using the comparative C_T_ method 2^−ΔCt^.

### Protein expression analysis in temozolomide-resistant AtT20 cells

To evaluate protein expression levels of Mgmt in AtT20 cells, Western blot analysis was performed on vehicle (DMSO)- or TMZ-challenged cells after each exposure. In addition, to confirm that the TMZ-resistant cells were stable, after 5 weeks of cell culture without drug treatment, vehicle- and TMZ-challenged cells were also collected to assess Mgmt protein expression.

Protein extraction was performed using radioimmunoprecipitation assay (RIPA) buffer (Thermo Scientific) containing HALT protease and phosphatase inhibitor and EDTA. An Advanced protein assay (catalog No. ADV01, Cytoskeleton Inc) was used to measure protein concentration. After protein denaturation, the samples were loaded on a sodium dodecyl sulfate–polyacrylamide gel electrophoresis gel, separated by electrophoresis, and transferred to a nitrocellulose membrane. The membrane was blocked for 2 hours with nonfat milk and followed by overnight incubation at 4 °C with primary antibody against Mgmt and incubation for 1 hour with secondary antibody for fluorescent detection.

Mgmt immunocytochemistry on vehicle-treated control and TMZ-resistant AtT20 cells (cultured on chamber slides) was performed using a primary goat antimouse MGMT antibody and a horseradish peroxidase–conjugated rabbit antigoat antibody. The method of cell preparation for immunocytochemistry, visualization of bound antibodies, and analysis of Mgmt positivity has been described previously (30). Digital analysis of Mgmt immunoreactivity and determination of the percentage of Mgmt-positive cells were performed using CellProfiler software, as described previously (31). Antibodies used were goat anti-Mgmt (RRID:AB_2143209); donkey antigoat immunoglobulin G (RRID:AB_621846); mouse antiactin (RRID:AB_476730); and goat antimouse immunoglobulin G (RRID:AB_2535724). Supplementary Table S3 shows the details of antibodies and dilutions that were used (24).

### Bisulfite polymerase chain reaction and pyrosequencing

Pyrosequencing of bisulfite-converted DNA was performed in vehicle- and TMZ-challenged cells in both protocols after each TMZ/vehicle challenge and at the end of the study period. To assess the methylation status of *Mgmt*, in total 10 CpGs sites located in 2 CpG islands at positions −8 to −250 (named *Mgmt*1) and −270 to −480 (named *Mgmt*2) from the transcription start site were analyzed (ENSMUSG00000054612).

As previously described (32), the following protocol was performed: First, we isolated DNA using the Wizard Genomic DNA Purification Kit (Promega). The genomic DNA was treated with sodium bisulfite using the EpiTect Plus DNA Bisulfite Kit (Qiagen) according to the manufacturer's recommendations. Subsequently, bisulfite-converted DNA was used for PCR reaction. For pyrosequencing, streptavidin-coated Sepharose beads (GE Healthcare) were used to bind PCR products and the samples were prepared using PyroGold SQA reagents (Qiagen) following the manufacturer's protocol. The analyses were performed using the Pyromark Q24 (Qiagen) system. Primers are listed in Supplementary Table S4 (24).

### Statistical analysis

We used analysis of variance or *t* test for numerous comparisons between groups in cell culture experiments. Nonlinear regression was used to calculate the half maximal response (EC_50_) of the effect of TMZ. Comparisons between vehicle-challenged cells and TMZ-challenged cells were performed using 2-way analysis of variance and Bonferroni as a post hoc test. To improve statistical power, in the analysis of MGMT expression in corticotroph tumors, the scores 0 (negative) and 1 (1%-25%) were combined, forming the group low MGMT expression (≤ 25%), and the scores 2, 3, and 4 were combined, forming the group moderate-high MGMT expression (>25%). To test the association between MGMT expression and clinical characteristics, Pearson chi-square was used for categorical variables, and *t* test for continuous variables. *P* values less than .05 were considered statistically significant. GraphPad Prism 5.0, and IBM SPSS Statistics, version 28.0.1.0, were used. Data are represented by mean ± SD.

## Results

### Heterogeneity of methylguanine-DNA methyltransferase immunohistochemistry in corticotroph tumors

MGMT immunostaining was heterogeneously distributed in 80% of corticotroph tumors (20/25). Areas with strong immunostaining were observed in contrast with others with less intense or even negative immunostaining within a tumor (Fig. 2). Among 25 ACTH tumors, 5 tumors were completely MGMT negative and 6 tumors presented with low (1%-25% positive cells, score 1) positivity, while 6 tumors showed the most intense MGMT staining (>75% positive cells), classified as score 4. The remaining 8 tumors presented with intermediate MGMT (26%-75% positive cells, scores 2 and 3) expression by IHC. Supplementary Table S1 summarizes the data of all individual cases (24).

**Figure 2:**
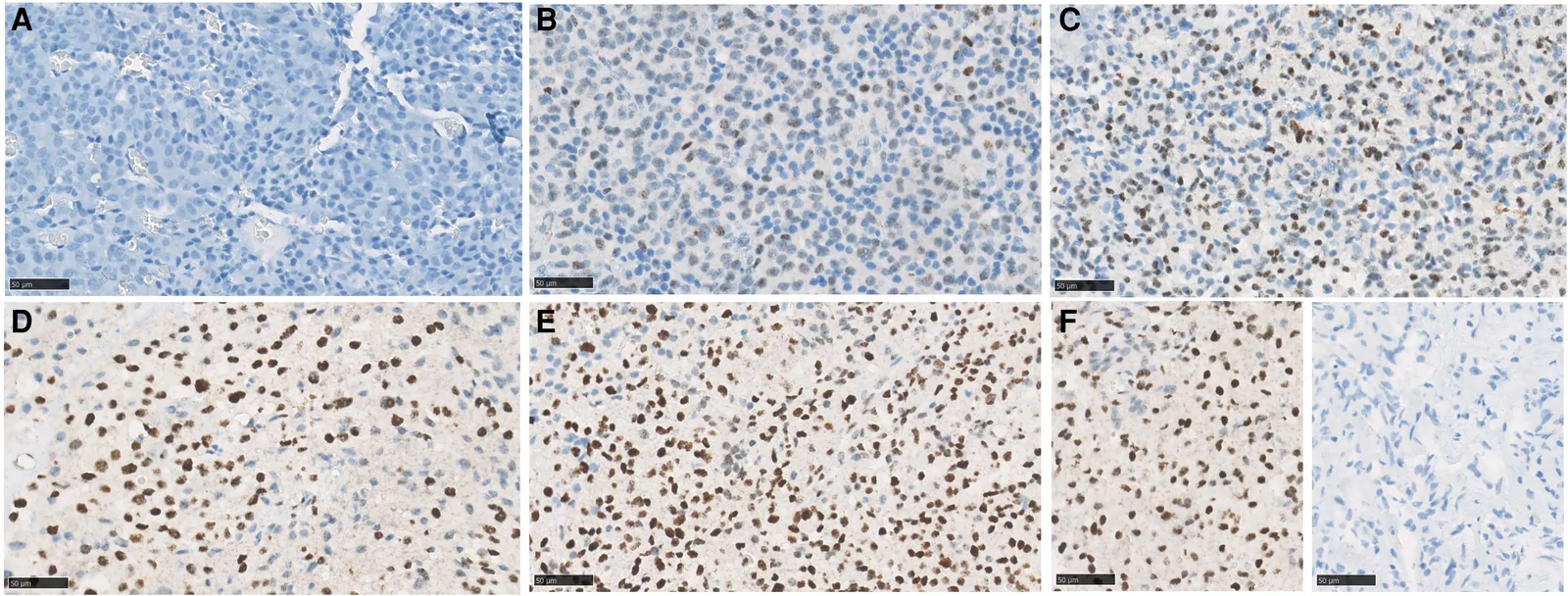
Methylguanine-DNA methyltransferase (MGMT) immunostaining in corticotroph tumors. A, Nonaggressive macroadenoma (case 11), score: 0; B, nonaggressive macroadenoma (case 14), score: 1; C, nonaggressive macroadenoma (case 9), score: 2; D, aggressive macroadenoma (case 6), score: 3; E, nonaggressive microadenoma (case 21), score: 4; F, nonaggressive microadenoma (case 22), case with heterogeneous expression of MGMT; left panel MGMT positive part, right panel MGMT negative part. Overall score: 2. Scale bar (100 µm) is shown in F. Four-tiered scale of MGMT staining: 0: negative, 1: 1% to 25%, 2: 26% to 50%, 3: 51% to 75%, and 4: >75%, according to the fraction of positive nuclear immunostaining.

### Methylguanine-DNA methyltransferase expression concerning clinical parameters

The corticotroph tumors were classified according to their clinical behavior into aggressive and nonaggressive. Six out of 25 tumors were considered aggressive, and most of these (66%, 4 tumors) had negative-low MGMT staining (<25% positive cells, scores 0 and 1). The majority of nonaggressive tumors (63%, 12/19 tumors) showed intermediate-high MGMT immunostaining (>25% of MGMT-positive cells) (*P* = .35). Clinical parameters are described in Table 1. In addition, 9 tumors were classified as microadenomas (<10 mm) and 16 as macroadenomas (≥10 mm). Nine (56%) of the macroadenomas had negative-low MGMT staining (<25%), while only 2 microadenomas (22%) had MGMT staining less than 25% (*P* = .2). Interestingly, tumors from patients who experienced persistent and/or recurrent disease after surgery had statistically lower MGMT expression compared to patients in remission after surgery (*P* = .008). Patients who required RT for disease control also had lower MGMT expression than those who did not need this treatment modality (*P* = .012). In contrast, no statistical difference in MGMT expression was identified between patients who required reoperation and those who did not (*P* = .18). In addition, tumors from challenging cases in which bilateral adrenalectomy was performed had statistically lower MGMT expression compared to cases in which this treatment modality was not necessary (*P* = .026) (see Table 1). Of note, in 2 patients, the tumor samples used to assess MGMT expression were obtained after bilateral adrenalectomy; in 1 case, the transsphenoidal surgery was performed 2 months after adrenalectomy, and in the other case, it was performed 2 years later due to tumor progression (Nelson syndrome).

**Table 1:** Correlation between methylguanine-DNA methyltransferase immunostaining and clinical parameters

		MGMT expression		
Characteristics	Total cohort (n = 25)	Negative-low (≤25%) (n = 11)	Moderate-high (>25%) (n = 14)	*P*
Age at diagnosis, mean ± SD, y	46.64 ± 8.61	44.45 ± 8.31	48.36 ± 8.75	.27
Sex, n (%)				.07
Female	22	8 (36.4%)	14 (63.6%)	
Male	3	3 (100%)	0 (0%)	
Tumor size, n (%)				.2
Microadenomas	9	2 (22.2%)	7 (77.8%)	
Macroadenomas	16	9 (56.3%)	7 (43.8%)	
Tumor behavior, n (%)				.35
Nonaggressive	19	7 (36.8%)	12 (63.2%)	
Aggressive	6	4 (66.7%)	2 (33.3)	
ACTH, median, min-max, pmol/L	14.7 (4.2-92)	13.4 (4.6-58.4)	14.9 (4.2-92)	.8
Severity of CD, n (%)				.3
Mild	12	4 (33.3%)	8 (66.7%)	
Moderate-severe	13	7 (53.8%)	6 (46.2%)	
Hypertension, n (%)				1
Yes	17	8 (47.1%)	9 (52.9%)	
No	8	3 (37.5%)	5 (62.5%)	
Diabetes, n (%)				.69
Yes	13	5 (38.5%)	8 (61.5%)	
No	12	6 (50%)	6 (50%)	
Tumor invasiveness, n (%)				.24
Noninvasive	15	5 (33.3%)	10 (66.7%)	
Invasive	10	6 (60%)	4 (40%)	
Persistent/recurrent disease after surgery, n (%)				.**008**
Yes	13	9 (69.2%)	4 (30.8%)	
No	12	2 (16.7%)	10 (83.3%)	
Reoperation, n (%)				.18
Yes	7	5 (71.4%)	2 (28.6%)	
No	18	6 (33.3%)	12 (66.7%)	
No. of surgeries, median (min-max)	1 (1-4)	1 (1-2)	1 (1-4)	.24
Radiotherapy, n %				.**012**
Yes	12	9 (75%)	3 (25%)	
No	12	2 (16.7%)	10 (83.3%)	
NA	1			
Bilateral adrenalectomy, n (%)				.**026**
Yes	4	4 (100%)	0 (0%)	
No	21	7 (33.3%)	14 (66.7%)	

Severity of CD was based on UFC, with mild hypercortisolism defined as a UFC level less than 2 times the ULN and moderate-high hypercortisolism as a UFC level above 2 times the ULN. *P*-values shown in bold indicate statistical significance (*P* < .05).

Abbreviations: ACTH, adrenocorticotropin; CD, Cushing disease; max, maximum; min, minimum; MGMT, methylguanine-DNA methyltransferase; NA, not available; RT, radiotherapy; UFC, urinary free cortisol; ULN, upper limit of normal.

Among the 6 tumors classified as aggressive, 2 (patients 4 and 6) were treated with TMZ. In patient 4, the tumor MGMT expression was between 1% and 25% (score 1). After undergoing surgery, RT, and subsequently bilateral adrenalectomy, the patient received 9 cycles of TMZ treatment, which stabilized the tumor growth. To date, 4 years after TMZ treatment, the tumor has remained stable. In patient 6, the tumor MGMT expression was between 51% and 75% (score 3). The patient underwent 2 neurosurgeries (transsphenoidal and transcranial), followed by RT. Due to continued tumor growth, TMZ was administered. In total, the patient received 5 cycles with an initial good response, evidenced by tumor stabilization for about 18 months. Then, the tumor regrew, and a second course of TMZ treatment was attempted, which was unsuccessful.

### AtT20 cell line with acquired temozolomide resistance

First, the dose-dependent effect of TMZ on growth of the murine corticotroph AtT20 cell line was determined. TMZ strongly reduced cell proliferation in a concentration-dependent manner. The TMZ EC_50_ was 20 µM (95% CI, 18-21 µM) (Fig. 3).

**Figure 3:**
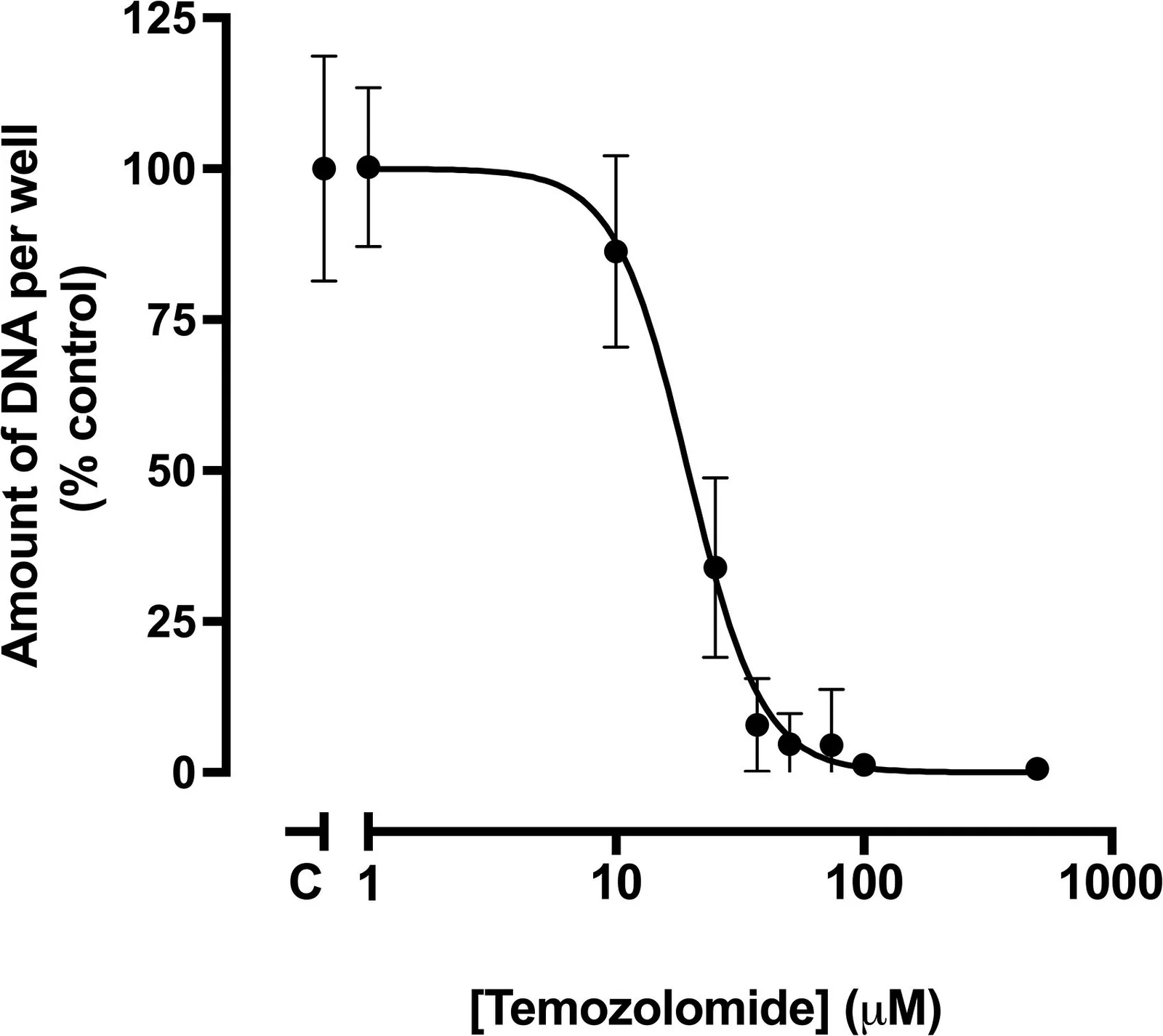
Dose-dependent effect of temozolomide (TMZ) on AtT20 cell proliferation. Dose-response curve of TMZ on AtT20 cell line. Cells were exposed to TMZ for 7 days, Half maximal response value is 20 µM (95% CI, 18-21 µM). Data are presented as mean ± SD from 4 independent experiments in quadruplicate. C on the x-axis represents vehicle-treated control. TMZ concentrations ranged from 1 to 500 µM as follows: 500 µM, 100 µM, 75 µM, 50 µM, 37.5 µM, 25 µM, 10 µM, and 1 µM.

Following the high TMZ_ch protocol, the AtT20 cell line rapidly became highly TMZ resistant after the first TMZ challenge with 100 µM TMZ (5 times EC_50_), with a statistically significant increase in EC_50_ in TMZ-challenged cells compared to vehicle-challenged cells, which was maintained after the third challenge with 300 µM TMZ (Table 2). In the TMZ low_ch protocol, the AtT20 cell line became only slightly resistant after the second TMZ challenge with 40-µM TMZ, with a progressive increase in resistance after the third and fourth challenges (Table 3). Tables 2 and 3 also show the fold-resistance values of TMZ-challenged, compared to vehicle-challenged cells.

**Table 2:** Half maximal response (EC_50_) of temozolomide (TMZ) in AtT20 cell line related to high TMZ-challenge protocol

Vehicle/TMZ challenges	Vehicle-challenged	TMZ-challenged	Fold resistance	*P*
	EC_50_, µM	EC_50_, µM		
	(95% CI)	(95% CI)		
**1st challenge**	33.9	173.0	5.1	<.001
	(28.5-40.3)	(149.4-200.4)		
**2nd challenge**	24.1	131.7	5.5	<.001
	(22.5-25.8)	(98.88-175.3)		
**3rd challenge**	19.4	123.0	6.3	<.001
	(17.4-21.7)	(106.7-141.7)		
**5 wk after**	29.8	189.0	6.3	<.001
**3rd challenge**	(27.6-32.3)	(155-230.4)		
**9 wk after 3rd challenge**	26.7	179.5	6.7	<.001
	(23-31.1)	(119.8-268.9)		

Treatment for the high TMZ-challenge protocol: first challenge with TMZ at a dose of 100 µM for 24 hours, followed by a drug-free period for 4 weeks. Second challenge with TMZ at a concentration of 200 µM for 24 hours, followed by a drug-free period of 3 weeks. The last challenge consisted of TMZ dose of 300 µM for 24 hours.

Abbreviations: EC_50_, half maximal response; TMZ, temozolomide.

**Table 3:** Half maximal response of temozolomide (TMZ) in AtT20 cell line related to low TMZ-challenge protocol

Vehicle/TMZ challenges	Vehicle-challenged	TMZ-challenged	Fold resistance	*P*
	EC_50_, µM	EC_50_, µM		
	(95% CI)	(95% CI)		
**1st challenge**	23.3	24.0	1.0	.508
	(19.8-27.5)	(21-27.5)		
**2nd challenge**	21.9	31.9	1.4	<.001
	(18.6-25.9)	(29.2-34.8)		
**3rd challenge**	36.9	57.1	1.7	<.001
	(34.5-39.5)	(49.4-66.1)		
**4th challenge**	20.6	69.3	3.4	<.001
	(16.8-25.3)	(62.1-77.3)		
**5 wk after 4th challenge**	26.1	61.7	2.4	<.001
	(22.3-30.4)	(51.9-73.4)		
**9 wk after 4th challenge**	21.6	75.9	3.5	<.001
	(17.3-26.9)	(67.3-85.6)		

Treatment for the low TMZ-challenge protocol: first challenge with TMZ at a dose of 20 µM for 24 hours, followed by a drug-free period for 2 weeks. The following reexposures to TMZ and drug-free periods were performed in the same manner with TMZ concentrations of 40 µM (second challenge), 60 µM (third challenge), and 80 µM (fourth challenge).

Abbreviations: EC_50_, half maximal response; TMZ, temozolomide.

Using the regular TMZ schedule (200 mg/m^2^/day) for CD patients, pharmacokinetic studies demonstrated that the peak of TMZ plasma concentration ranges from 50 to 72 µM (28). We observed highly and partially resistant cells using these TMZ concentrations in the high and low TMZ_ch protocols, respectively (Fig. 4A and 4B). In the dose-response experiment performed 3 weeks after the third challenge, in TMZ-challenged cells the maximum clinically achievable dose of 75 µM had a minor effect (28% of inhibition) on cell proliferation in the high TMZ_ch protocol (see Fig. 4A). In comparison, it inhibited cell proliferation by about 50% in the low TMZ_ch protocol in the experiment conducted 2 weeks after the fourth challenge (see Fig. 4B). The same concentration of TMZ completely blocked cell proliferation in the vehicle-treated control cells.

**Figure 4:**
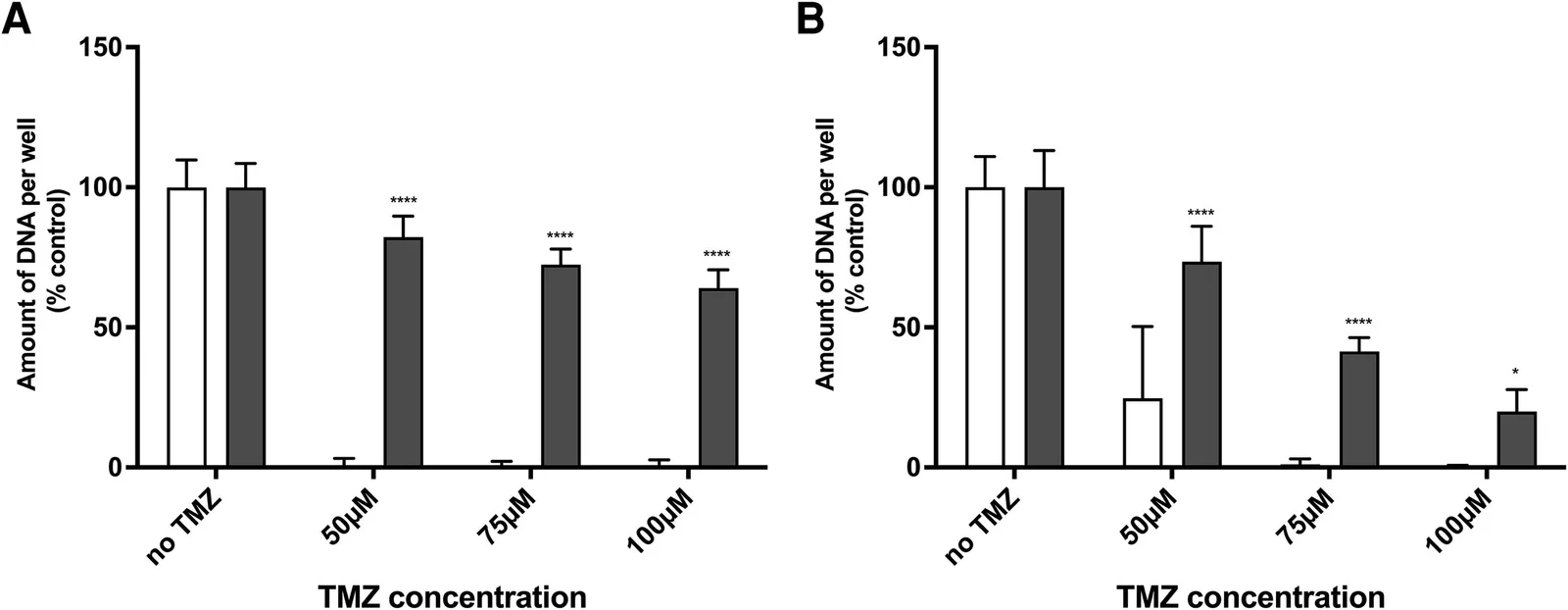
Effect of temozolomide (TMZ) on AtT20 cell proliferation. Effect of TMZ on AtT20 cell line proliferation demonstrated by the percentage of DNA amount in vehicle- and TMZ-challenged cells in A, high TMZ_ch protocol and B, low TMZ_ch protocol. White bars represent vehicle-challenged cells. Gray bars represent TMZ-challenged cells. ****P less than .0001 and *P less than .05. Data are presented as mean ± SD in quadruplicate. In the A, high_ch protocol, the dose-response experiment was performed 3 weeks after the third challenge, while in B, the low TMZ_ch protocol, the experiment was conducted 2 weeks after the fourth challenge. Both 7-day experiments were performed using increasing concentrations of TMZ, ranging from 1 to 500 µM (500, 100, 75, 50, 37.5, 25, 10, and 1 µM); results obtained with TMZ doses of 500, 37.5, 25, 10, and 1 µM are not shown.

To investigate if the cells would maintain their TMZ-resistant profile, we performed dose-response experiments at 5 and 9 weeks of the last challenge in the low and high TMZ_ch protocols. These experiments consisted of a 7-day incubation with increasing concentrations of TMZ (range, 1-500 µM), as described in the Materials and methods section. We observed that the TMZ-challenged cells remained resistant compared to vehicle-challenged cells in both protocols (see Tables 2 and 3).

### Cell cycle analysis in vehicle- and temozolomide-challenged cells

In vehicle-challenged cells, TMZ treatment induced changes in all stages of the cell cycle. There was a strong accumulation of TMZ-treated cells in the G2/M phase and a reduction in the G0/G1 phase (Fig. 5A and 5C).

**Figure 5:**
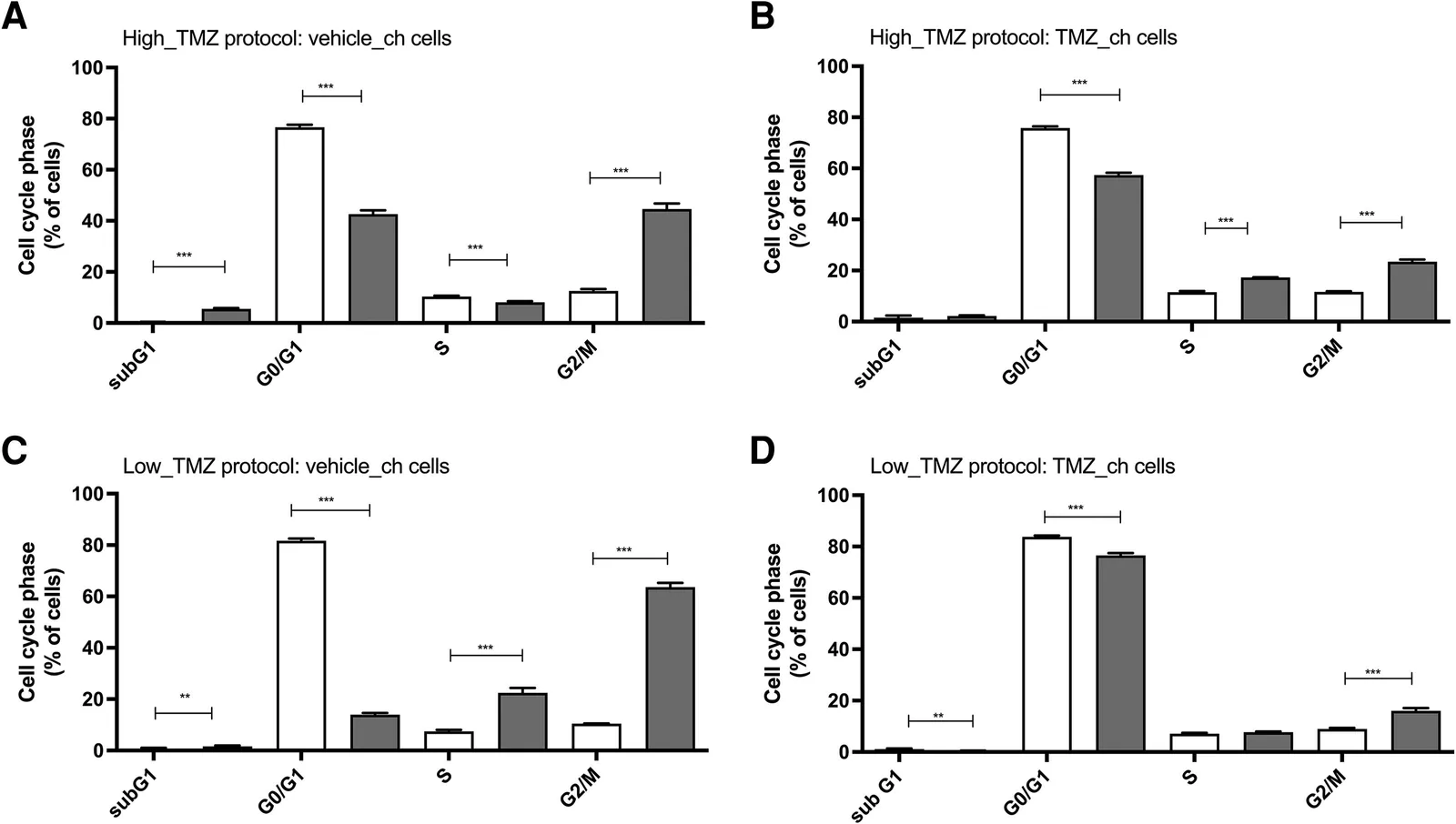
Cell cycle progression in vehicle and temozolomide (TMZ)-challenged cells. Stages of cell cycle in vehicle and TMZ-challenged cells with TMZ treatment compared to cells with no drug incubation; A, In the high TMZ-challenge protocol, vehicle-challenged cells were treated with vehicle (light gray bars) or TMZ at dose of 100 µM (white bars); B, In the high TMZ-challenge protocol, TMZ-challenged cells were treated with vehicle (light gray bars) or TMZ at dose of 100 µM (white bars); C, In the low TMZ challenge protocol, vehicle-challenged cells were treated with vehicle (light gray bars) or TMZ at dose of 80 µM (white bars); D, In the low TMZ challenge protocol, TMZ-challenged cells were treated with vehicle (light gray bars) or TMZ at dose of 80 µM (white bars). Data are expressed as the mean ± SD (3 replicates per condition). **P less than .008 and *** *P* less than .0001.

Incubation with TMZ in TMZ-challenged cells (from both high and low TMZ-challenged protocols) resulted in only a small effect in phases of cell cycle compared to vehicle-treated cells. There was a slight accumulation of treated cells in the G2/M phase, and a reduction of treated cells in the G0/G1 phase compared to cells without drug treatment (Fig. 5B and 5D).

### Expression of *Mgmt* and mammalian mismatch repair (*Msh2*, *Msh6*, *Pms2*, and *Mlh1*) messenger RNA in vehicle-and temozolomide-challenged cells

TMZ challenges resulted in a strong increase in *Mgmt* in TMZ-challenged cells compared to vehicle-challenged cells (Fig. 6A and 6B). In both protocols there was a gradual increase in *Mgmt* expression throughout each challenge of TMZ. While in the TMZ high_ch protocol a statistically significant increase was already observed after the first challenge, in the TMZ low_ch protocol the *Mgmt* expression was not increased until the third TMZ challenge. This finding is consistent with the results of resistance to the cell growth–inhibitory effects of TMZ.

**Figure 6:**
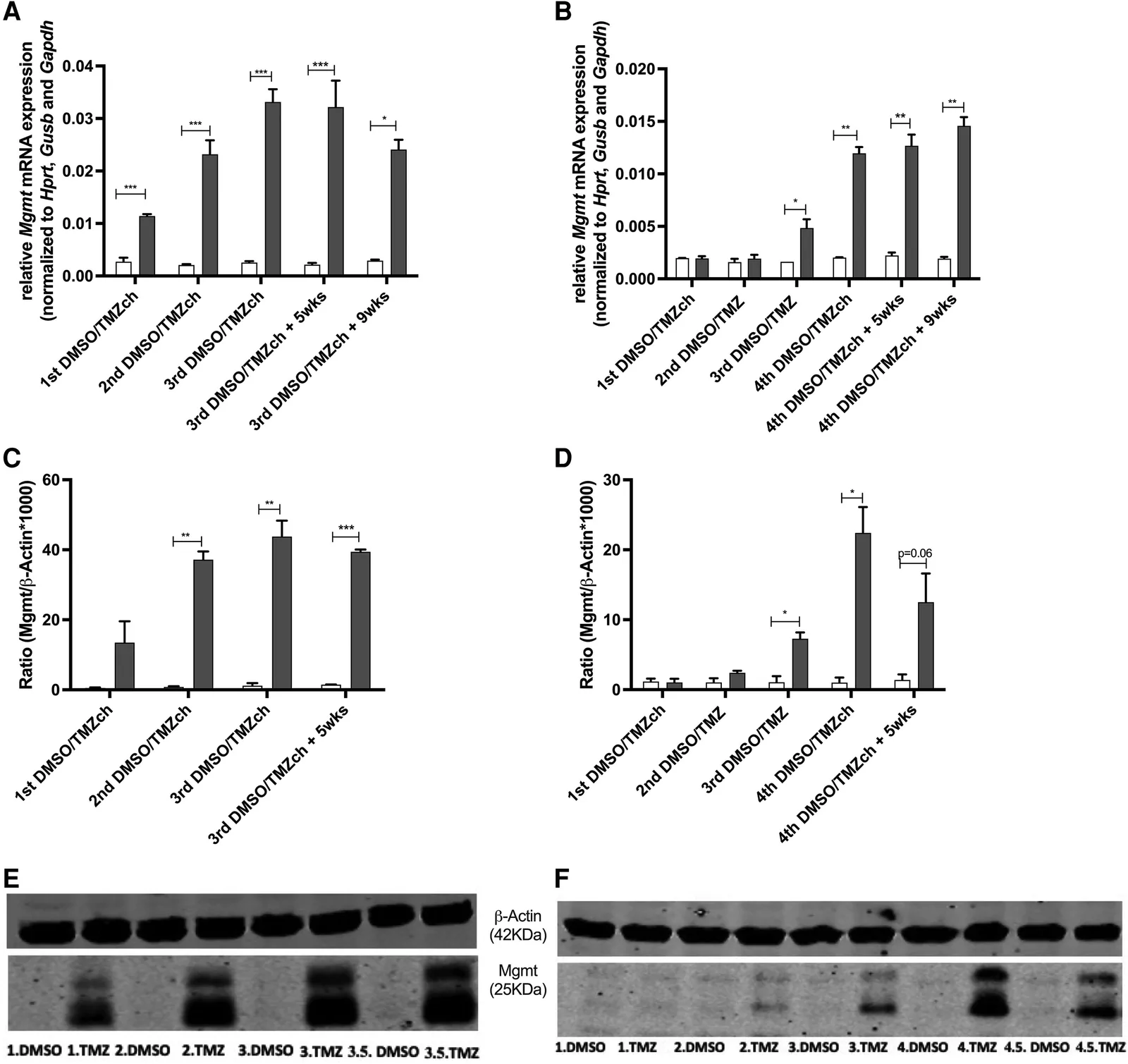
Methylguanine-DNA methyltransferase (*Mgmt*) messenger RNA (mRNA) and protein expression. A, *Mgmt* mRNA expression in the high temozolomide (TMZ)-challenge protocol; B, *Mgmt* mRNA expression in the low TMZ-challenge protocol; C, Densitometric analysis of Mgmt protein expression in the high TMZ-challenge protocol; D, Densitometric analysis of Mgmt protein expression in the low TMZ-challenge protocol; E, Images of Western blot analysis in the high TMZ-challenge protocol; F, Images of Western blot analysis in the low TMZ-challenge protocol. Western blot images are displayed by alternating vehicle-challenged with TMZ-challenged samples: first dimethyl sulfoxide (DMSO)/TMZ challenge (No. 1), second DMSO/TMZ challenge (No. 2), third DMSO/TMZ challenge (No. 3), fourth DMSO/TMZ challenge (No. 4, in TMZ low_ch protocol), 5 weeks after the last challenge (No. 3.5, in TMZ high_ch protocol and No. 4.5, in TMZ low_ch protocol). White bars represent vehicle-challenged cells. Gray bars represent TMZ-challenged cells. Values represent mean ± SD (2 replicates per condition). *P less than .05; **P less than .01; ***P less than .001.

TMZ challenges did not induce major changes in mRNA expression of the different MMR components (data not shown).

Expression of Mgmt protein in vehicle- and temozolomide-challenged cells

Mgmt protein expression mirrored the results of *Mgmt* mRNA. In both protocols, Western blot results revealed a gradual increase in Mgmt protein expression with each TMZ challenge. In the TMZ high-ch protocol, the increase of Mgmt expression was already detected after the first challenge, while in TMZ low-ch protocol an increase was observed only after the third TMZ challenge, reaching its maximum after the fourth TMZ challenge. The higher expression of Mgmt protein in TMZ-challenged cells compared to vehicle-challenged cells was seen even after 5 weeks of the last TMZ/vehicle challenge (Fig. 6C-6F and Supplementary Fig. S1 for full Western blot images) (24).

### *Mgmt* promoter methylation in vehicle and temozolomide-challenged cells

In the *Mgmt* promoter, the mean percentages of methylation in each CpG were high, ranging from 40% to 100% in both vehicle- and TMZ-challenged cells. The pyrosequencing results were strongly concordant between vehicle-challenged and TMZ-challenged cells. No difference was observed in the level of methylation of each CpG throughout each vehicle/TMZ challenge (Supplementary Fig. S2) (24).

### Temozolomide challenges affect AtT20 cell morphology and the percentage of *Mgmt*-expressing cells

In vehicle-challenged cells morphologically 2 types of cells were distinguished by light microscopy: small, rounded cells and fusiform-shaped cells. In TMZ-challenged cells only rounded cells were detected in culture (Fig. 7A and 7B). It is apparent that TMZ challenges may have affected AtT20 cell morphology by selecting a cell population type, which was evident only in the high TMZ_ch protocol. In the low TMZ_ch protocol TMZ-challenged cell morphology was comparable to that of vehicle-challenged cells (not shown). Similarly, immunocytochemical analysis (Fig. 7C and 7D) showed a statistically significant 1.8-fold increase (*P* < .05) in the number of nuclear Mgmt-expressing cells in acquired TMZ-resistant cells compared to vehicle-treated control cells (see Fig. 7C). There was almost no change in the nuclear Mgmt immunoreactivity per cell (see Fig. 7D). Again, this effect was observed only in the TMZ-resistant cells in the high TMZ_ch protocol.

**Figure 7:**
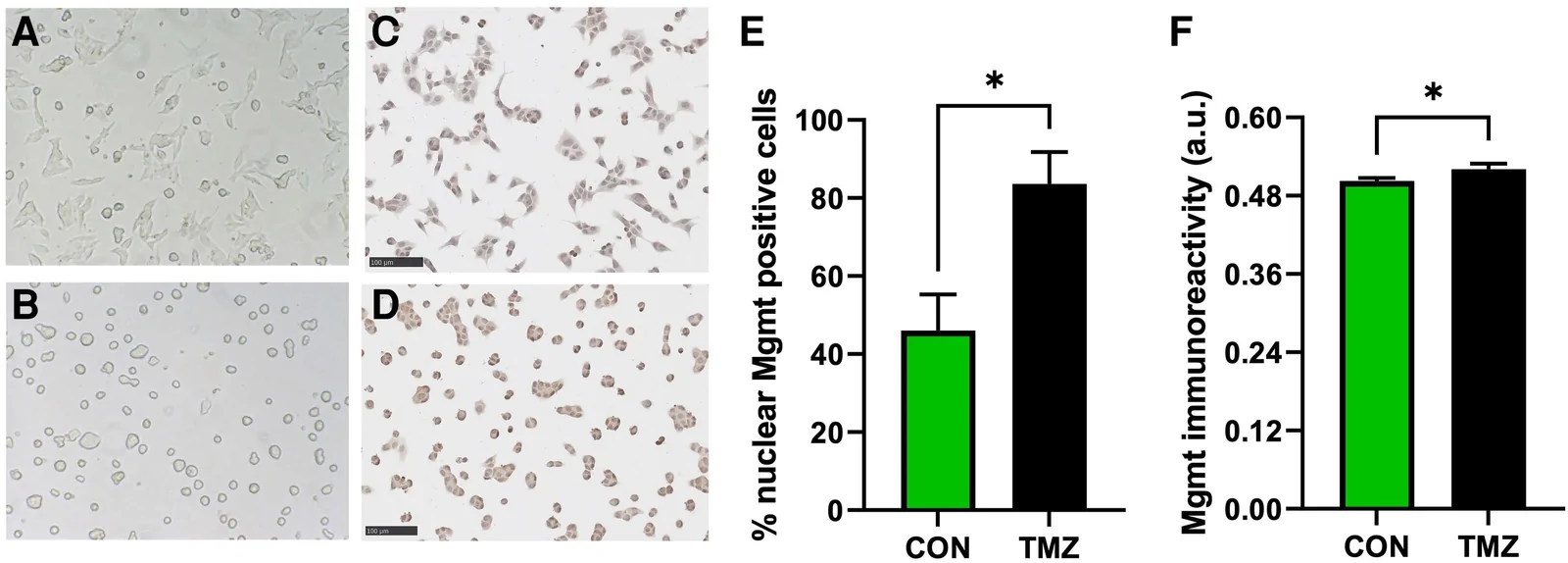
Vehicle and temozolomide (TMZ)-challenged AtT20 cell morphology and methylguanine-DNA methyltransferase (Mgmt) immunoreactivity in the high TMZ_ch protocol. A, In vehicle-challenged cells, 2 types of cell morphology were observed: a small rounded one and a fusiform-shaped one; B, in TMZ-challenged cells, only rounded cells were detected in culture; C, representative photomicrograph of Mgmt-positive cells in vehicle-treated control cells; D, representative photomicrograph of Mgmt-positive cells in TMZ-challenged cells; E, percentage of nuclear Mgmt-positive cells; F. Mgmt immunoreactivity in arbitrary units (a.u.). *P less than .05. Mgmt immunoreactivity per cell was determined in control, vehicle-treated cells, and cells that had developed resistance to TMZ following 3 TMZ pulse challenges. Analysis was performed using CellProfiler.

## Discussion

TMZ has improved the prognosis of patients with aggressive (corticotroph) pituitary tumors and carcinomas (2, 33-37). However, a substantial proportion of patients are resistant or develop resistance to the drug, which is the dominant reason for treatment failure (3, 4, 21, 34, 37, 38). Our results demonstrate a strong heterogeneous MGMT expression in human corticotroph tumors, which may provide the tumor cells with a mechanism for surviving TMZ treatment, especially subclones with high MGMT expression (39). Accordingly, in our in vitro AtT20 corticotroph tumor model, we showed that increased MGMT expression and an increase in the number of MGMT-positive cells were associated with acquired TMZ resistance.

We observed a strong heterogeneous distribution of MGMT immunostaining in most corticotroph tumors. It was observed that within the same tumor, the percentage of MGMT-positive cells varies widely, reflecting heterogeneous intratumoral MGMT expression and suggesting the presence of subpopulations with different MGMT expression status in individual tumors. In 1 striking case, we even found 2 distinct parts of the tumor exhibiting completely different MGMT expression patterns; 1 part was completely negative, and the other strongly positive for MGMT staining. The heterogeneous pattern of MGMT expression observed in the majority of corticotroph tumors could contribute to the selection of preexisting resistant clones (high MGMT expression) by TMZ treatment, thus leading to TMZ resistance.

MGMT is a DNA repair enzyme with a role in protecting cells from driver mutations. Loss of expression of this enzyme potentially results in the accumulation of genetic events, which may promote aggressive tumor behavior (40, 41). However, the association between low MGMT expression and tumor aggressiveness is controversial. Some studies have shown that low MGMT expression is associated with a higher recurrence rate, invasiveness, and aggressiveness, while others have failed to show such an association (19, 42-47). A previous study, which included only corticotroph tumors, reported an association between low MGMT expression by IHC and larger tumors, a lower frequency of disease remission, and increased tumor aggressivity (48). Although we did not find an association between low MGMT and tumor size or tumor invasiveness in the present study, we confirmed that low levels of MGMT expression (<25% positive cells) were associated with persistent and/or recurrent disease after transsphenoidal surgery. Furthermore, the majority of the aggressive ACTH-secreting tumors (66%) had low MGMT IHC expression, although it was not statistically significant.

In the in vitro part of the present study, TMZ inhibited cell proliferation of a corticotroph pituitary tumor cell line by inducing cell cycle arrest. Additionally, TMZ inhibited the number of cells in a dose-dependent manner. The EC_50_ of the inhibitory effect of TMZ was 20 µM, which is close to the clinically achievable plasma concentration with current TMZ treatment for pituitary tumors (range, 50-72 µM) (27, 28). These results are in line with in vitro studies demonstrating an inhibitory effect of TMZ in pituitary adenoma cell lines (49, 50) Clinically, in approximately half of the initial responders to TMZ treatment, there is recurrence of the disease, and 60% to 90% of these patients appear to have acquired resistance to a subsequent course of TMZ treatment (3-5, 7-9).

To better understand the acquired TMZ resistance, we developed 2 models using a corticotroph tumor cell line to understand the mechanism leading to acquired TMZ resistance. In the high-level TMZ_ch protocol, we challenged the cells with increasing high doses of TMZ (100-200-300 μM), which resulted in a stable and highly TMZ-resistant cell line. Conversely, in the low-level protocol we challenged the cells using increasing TMZ concentrations (20-40-60-80 μM) that can be considered clinically relevant given that the serum concentration of TMZ in patients ranges from 50 to 70 μM after a standard TMZ schedule (150-200 mg/m^2^ for 5 days) (5, 27). TMZ-induced resistance was demonstrated by a sustained increase in TMZ EC_50_ and by lower changes in cell cycle induced by TMZ in both protocols.

To assess potential mechanisms for such acquired resistance to TMZ, we analyzed the expression of MGMT, which modulates the response to TMZ. After TMZ challenges, we observed a marked increase in MGMT expression in TMZ-resistant cells, both at the mRNA and protein levels in whole-cell lysates, as well as in the number of MGMT-positive cells, as determined by IHC. In contrast, by IHC, no major change in the amount of MGMT immunoreactivity per cell was observed. Factors other than MGMT also modulate the antitumoral effect of TMZ. The role of the MMR system in alkylating agent resistance has been previously reported (13, 14). However, in the present study, there was no relevant change in MMR expression levels in either protocol. Therefore, our data suggest that overexpression of MGMT, rather than altered MMR expression, is the main factor associated with TMZ resistance.

Previous clinical and in vitro studies of pituitary tumors found an association between high expression of MGMT and low sensitivity to initial treatment with TMZ (primary resistance) (2, 9, 18, 21, 49, 50). Our data show this mechanism in the context of acquired resistance to this drug after the initial TMZ challenge (secondary resistance). Interestingly, in the clinically relevant low TMZ_ch protocol, after each challenge, there was a gradual increase in resistance accompanied by increasing levels of MGMT expression. Moreover, high MGMT expression in TMZ-challenged cells was maintained until the end of the study period, resulting in a stable resistant phenotype.

One potential mechanism of acquired chemoresistance is TMZ-induced epigenetic changes in tumor cells (51). Although we consistently observed a gradual increase in MGMT expression after each TMZ challenge, no difference was seen in the methylation status of the *MGMT* gene, suggesting that another mechanism is involved in TMZ-induced MGMT expression.

Selection of a cell population with high MGMT expression may be considered as a potential mechanism of acquired resistance. We observed a clear change in cell morphology when cells became TMZ resistant. In high-dose TMZ_ch–resistant cells, only round-shaped cells, rather than round and fusiform-shaped cells, were observed, as well as a strong increase in the percentage of MGMT-expressing cells with no change in the amount of immunoreactive MGMT per cell, suggesting selective pressure by TMZ treatment.

Based on the present findings, it can be hypothesized that TMZ treatment might select clones of resistant cells with high MGMT expression, which can, in turn, give rise to recurrent tumors unresponsive to a second course of TMZ (1, 4, 21).

Our study has some limitations. First, our data were acquired using only one type of tumor cell line: AtT20 mouse corticotroph tumor cells. To the best of our knowledge, however, there are no other relevant corticotroph tumor cell line models available. Additionally, we evaluated MMR component expression at the mRNA but not protein level. We aimed to select equal numbers of microadenomas and macroadenomas, and to include all available aggressive tumors, which may introduce selection bias. We determined MGMT expression in corticotroph tumors naive to TMZ treatment. Spatial transcriptomics may also provide additional insight into intratumor heterogeneity, which warrants further investigation in future studies. Ideally, to confirm TMZ-inducing selective pressure of MGMT clones, it would be interesting to evaluate MGMT expression before and after TMZ treatment in corticotroph tumors. Realistically, obtaining such tumors was not feasible. Therefore, we first demonstrated subclones with different levels of MGMT expression in corticotroph tumors; then, we used our in vitro model to support our hypothesis that TMZ treatment can induce selective pressure, resulting in high MGMT-expressing clones of cells.

In conclusion, we show a strong intertumoral and intratumoral heterogeneity of MGMT expression in corticotroph tumors. Our results suggest that MGMT can be considered in determining resistance to TMZ treatment in corticotroph tumor cells. We created an in vitro model of acquired TMZ resistance in a corticotroph adenoma cell line, reflecting the development of TMZ resistance after repeated TMZ treatment as observed in vivo. The induction of TMZ resistance was dose dependent and is, at least in part, related to increased MGMT expression, as well as an increase in the number of MGMT-positive tumor cells. Upregulation of MGMT expression was not mediated via modulation of the MGMT promotor methylation status. On this basis, it can be hypothesized that clonal selection of high MGMT-expressing cells is involved in this acquired resistance.

## Data Availability

Some or all datasets generated during and/or analyzed during the current study are not publicly available but are available from the corresponding author on reasonable request.

## Abbreviations

ACTH: adrenocorticotropic hormone
CD: Cushing disease
DMSO: dimethyl sulfoxide
EC_50_: half maximal response
high TMZ_ch: high TMZ challenge
IHC: immunohistochemistry
low TMZ_ch: low TMZ challenge
MGMT: methylguanine-DNA methyltransferase
MMR: mammalian mismatch repair
mRNA: messenger RNA
qPCR: quantitative polymerase chain reaction
RT: radiotherapy
TMZ: temozolomide
UFC: urinary free cortisol
ULN: upper limit of normal
